# Esophageal submucosal gland duct adenoma: a case report and literature review

**DOI:** 10.3389/fonc.2025.1525781

**Published:** 2025-02-06

**Authors:** Bing Zhou, Jian Zheng, Ling Huang, Hua Hao

**Affiliations:** ^1^ Department of Pathology, Second Affiliated Hospital of Jiujiang University, Jiujiang, China; ^2^ Department of Pathology, Yangpu Hospital, School of Medicine, Tongji University, Shanghai, China; ^3^ Department of Digestion Medicine, Yangpu Hospital, School of Medicine, Tongji University, Shanghai, China

**Keywords:** esophageal, esophagus diseases, EMR, CK7, CK5/6

## Abstract

**Introduction:**

Esophageal submucosal gland duct adenoma (ESGDA) is a rare, benign tumor with non-specific clinical features and imaging findings, often leading to misdiagnosis.

**Case report:**

In this report we describe the clinicopathological features of a new-onset case of ESGDA and review 19 previously-reported ESGDA cases in the literature.

**Results:**

The median age of the 20 patients was 70 years, and the male-to-female ratio was 5:2. Lesions located in the lower esophagus accounted for 81% of the cases. Chest and abdominal discomfort were the main clinical symptoms, and endoscopic findings mostly included polypoid masses that were impossible to correctly diagnose prior to excision and biopsy. On microscopic examination, the submucosal tumor was found to be composed of glandular cavities of various sizes, bland cytology with infrequent mitotic figures, and the interstitium was infiltrated by a large number of lymphocytes. Immunohistochemical analysis revealed the expression of adenomyoepithelial cytokeratins (CK) CK7, CK5/6, and p63. Local tumor resection was performed, the longest follow-up period was 132 months of the 20 patients, the prognosis was favorable, and no recurrence or metastasis.

**Discussion:**

ESGDA is more common in the lower esophagus in elderly patients, and its clinical symptoms are atypical. Taking into account its characteristic microscopic morphology and immunohistochemical markers, the possibility of this rare disease should be considered to avoid misdiagnosis or missed diagnosis. Complete en bloc resection with Endoscopicp may be the best strategy for both the diagnosis and treatment of this entity.

## Introduction

Esophageal submucosal gland duct adenoma (ESGDA) is a benign tumor that originates in the submucosal gland (SMG) of the esophagus and is unrelated to Barrett’s esophagus (1). To date, only 19 such cases have been reported in the literature worldwide. ESGDA has no specific clinical or endoscopic manifestations and is often misdiagnosed as one of many other types of benign and/or malignant diseases; however, its pathology includes characteristic changes. In this report, we discuss a case of ESGDA, and its clinicopathological features are analyzed in the context of a literature review on the subject, in order to improve the general understanding of this rare disease and provide a better basis for its future diagnosis and treatment.

## Case description

### Clinical data

**Figure 1:**
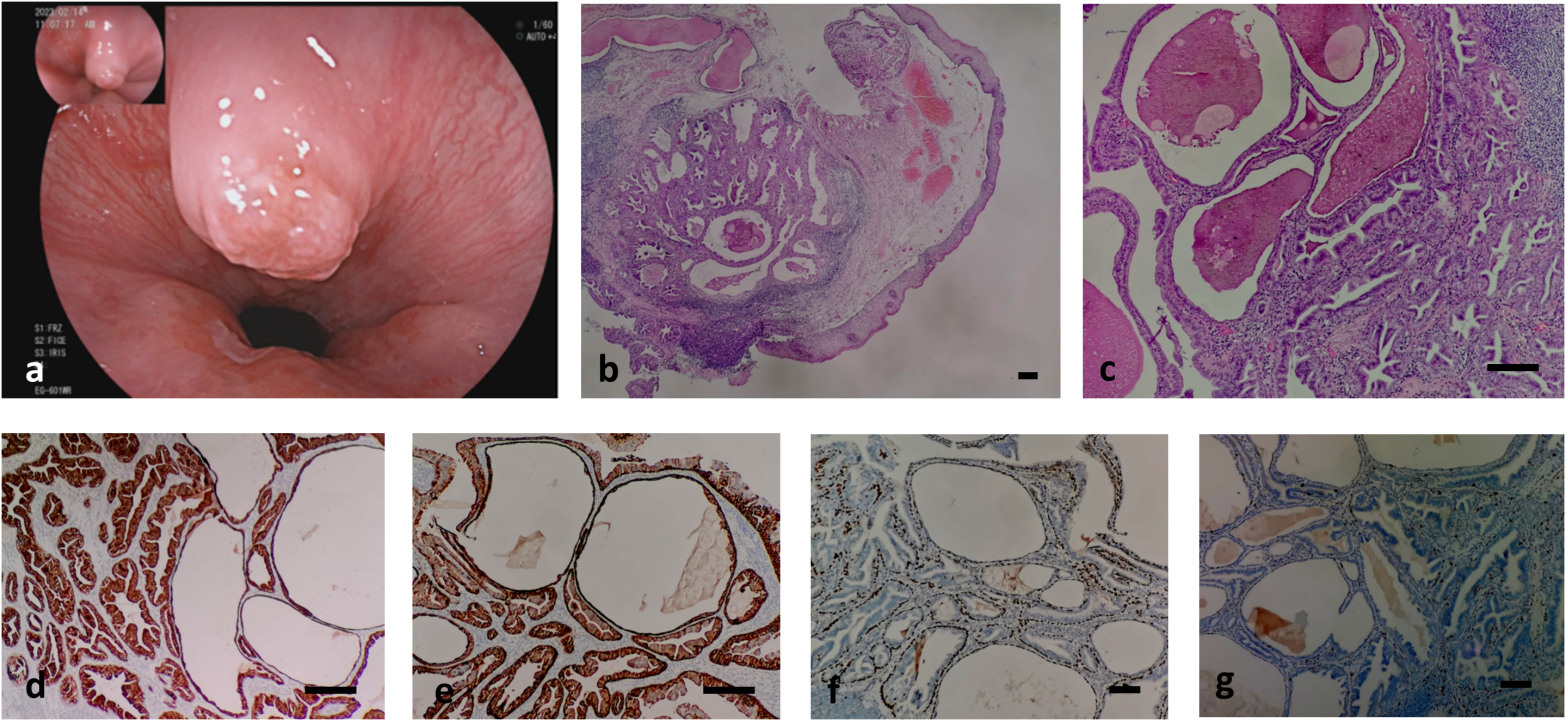
shows the endoscope image and pathological images of the case. **(A)** shows the mucosal bulge near the cardia of the esophagus; **(B, C)** shows the histology of the case. Low (b, hematoxylin-eosin, × 40) and intermediate (c, hematoxylin-eosin, × 200); **(D–G)** shows the Immunohistochemical staining of the case. **(D)** CK7-positive double-layered cells in ductal adenoma of esophageal submucosal gland, indicating glandular epithelial differentiation; **(E)** CK5/6 (+) cells in the inner layer and outer layer of ductal adenoma of esophageal submucosal gland indicating myoepithelial and squamous epithelial differentiation; **(F)** Positive p63 in the outer basal layer of ductal adenoma of esophageal submucosal gland, indicating myoepithelial differentiation; **(G)** proliferation index of esophageal submucosal ductal adenoma <1%, indicating have a good prognosis. Bar=50μm.

The patient was an 83-year-old woman who was admitted to the hospital due to intermittent left lower abdominal pain and abdominal distension lasting 1 week. She had a good appetite and no nausea, vomiting, diarrhea, black stools, or any other discomforts. At the time of writing this report, she is currently visiting our hospital for further diagnosis and treatment, and has been admitted to the outpatient department for abdominal distension. ESGDA is often accompanied by abdominal distension, discomfort, absence of hunger, pain, cough, expectorated sputum, acid reflux discomfort, fever, chills, black stool, sticky frozen stool, chronic constipation, and chronic high blood pressure (reported as high as 180/88 mmHg). In our case, gastroscopic examination showed venous tumor formation in the upper part of the esophagus (blue in color) and a mucosal bulge near the cardia at 11-12 o’clock of approximately 1.0 cm × 0.8 cm (**Figures 1A**). Its surface was uneven, suggesting fibrous tissue polyps. Endoscopic mucosal resection (EMR) was performed, and samples were sent for pathological examination.

### Pathological examination

A sample of the polypoid mass was sent for inspection. It measured approximately 0.7 cm × 0.7 cm × 0.3 cm, with a mucous membrane on the surface. The cut surfaces showed a well-circumscribed, encapsulated mass had a predominantly solid and gray-white appearance with cystic spaces. Microscopic examination under a low-magnification microscope revealed that the lesion was located in the submucosa and the boundary was relatively clear. The mucosal surface was lined with mild hyperplasia of the squamous epithelium, and lesions consisting of glandular or cystic cavities of various sizes, dilations, or hyperplasias were observed (**Figures 1B, C**). Red-stained unstructured secretions were observed in the gland and cyst cavities, and increased lymphocyte infiltration with lymphoid follicle formation was observed in the periphery of the lesion (**Figures 1B, C**). Atrophy of the submucosal acini was also observed. Under a high-power microscope, the lesion was found to be composed of double-layered cells. The inner-layer cells were glandular epithelial cells that were columnar, cubic, or flat, with eosinophilic cytoplasms and small nuclei located at the bases. The glandular epithelial cells were observed to be proliferating in multiple layers or forming papillary structures. The outer layer consisted of basal layer cells that were spindle-shaped, eosinophilic, sparse, or had transparent cytoplasm. The two layers of cells were mild in morphology, with no pathological mitotic figures and minimal atypia (**Figures 1B, C**).

### Immunophenotype

The inner-layer cells of the submucosal ductal adenoma were positive for cytokeratins (CK) CK7 (**Figures 1D**) and CK5/6 membrane or cytoplasm (**Figures 1E**). The basal-layer cells were positive for CK7 (**Figures 1D**), CK5/6 membrane or cytoplasm (**Figures 1E**), and p63 nuclei (**Figures 1F**). Staining for CK20, CDX2, MUC2, MUC5AC, and MUC6 were all negative, and the Ki-67 proliferation index was <1% (**Figures 1G**).

Pathological diagnosis: Esophageal submucosal gland duct adenoma (ESGDA).

### Treatment and follow-up

EMR was used for complete dissection of the polyps, and the patient’s postoperative recovery proceeded well. No recurrence was observed after 17 months of follow-up.

## Discussion

Esophageal adenomas are rare and are mostly secondary to Barrett’s esophagus caused by gastroesophageal reflux. Primary ductal adenomas typically originate in the esophageal SMG. Since the first report by Tsutsumi et al. (2) in 1990, only 19 cases have been reported, and its pathogenesis remains unclear. Some studies have shown that esophageal gland ductal cysts are similar to ESGDA in terms of clinical characteristics and tissue structure, and it has been speculated that they may be precursor lesions of ESGDA (3). Other scholars believe that the occurrence of ESGDA is related to the damage of the esophageal submucosa and infiltration of inflammatory cells. When the glandular duct is damaged, pluripotent stem cells proliferate in undirected ways, while inflammatory exudation and fibrous tissue hyperplasia repair lead to blockage of the duct opening, which in turn forms ESGDAs that are characterized by cysts with multilayered epithelia that form a papillary structure (4). Genetic mutation detection of ESGDA revealed BRAF V600E mutation similar to in sialadenoma papilliferum, providing further evidence that it has the same stem cell origin (5).

ESGDA only occurs in the esophagus, its incidence is low and its epidemiological characteristics remain unclear. In this study, a new ESGDA case was identified, and the clinicopathological features of the 19 cases previously reported in the literature were summarized (**Table 1**) (6–19). Among the 20 cases reported so far, there were 14 men and 6 women (male-to-female ratio, 5:2), the age of onset was 45–83 years, and the median age was 70 years. The clinical manifestations of ESGDA are mostly atypical, with upper abdominal discomfort being the most common, accounting for 45% of all the patients. When the tumor is large, there may be a feeling of choking when swallowing. Some patients may be asymptomatic and may only have their tumors discovered through endoscopy. The entire esophagus can be affected, but the condition is usually more concentrated in the SMG of the lower esophagus. Therefore, ESGDA is more common in the lower esophagus, accounting for 81% of the total lesions (20). Similar findings were observed in the present case, as the lesions were located in the lower esophagus. The tumors varied in size, ranging from 3 to 35mm in diameter with an average of 10.4mm. On endoscopy, most are seen as raised or polypoid tumors that are difficult to distinguish from other inflammatory fibrous polyps, leiomyomas, or lymphangiomas. All 20 cases of ESGDA that have been reported so far could not be correctly diagnosed prior to pathological analysis.

**Table 1:** Summary of the clinicopathological features of esophageal gland duct adenoma.

Author	Age /sex	Clinical manifestation	Site(esophagus)	Diameter (mm)	Provisional clinicaldiagnosis	Treatment	Follow-up months	Outcome
Tsutsumi et al (2)	77/M	Nausea	Mid	10	esophageal polyp	EP	24	AWD
Takubo et al (6)	58/M	Abdominal discomfort	Mid	8	polypoid tumor	Esophagectomy	6	AWD
Rouse et al (7)	81/M	Dysphagia	Distal	15	esophageal polyp	Polypectomy	12	AWD
Su et al (8)	70/M	Abdominal distention	Distal	10	polypoid tumor	Esophagectomy	12	AWD
Agawa et al (9)	71/M	NA	Distal	15	esophageal tumor	EP	12	AWD
Hayashi et al (10)	60/F	Abdominal discomfort	upper	11	submucosal tumor	EP	132	AWD
Chinen et al (11)	60/M	None	Distal	6	intramural mass	EP	NA	NA
Harada et al( 12)	75/M	NA	Distal	3	None	Total gastrectomy	NA	NA
Shibata et al (13)	66/F	None	Distal	5	leiomyoma or granular cell tumor	ESD	NA	NA
Nie et al (14)	74/M	Retrosternal discomfort	Distal	5	leiomyoma	EMR	54	AWD
Nie et al (14)	54/F	Abdominal discomfort	Distal	3	leiomyoma	EMR	48	AWD
Nie et al (14)	45/M	NA	Distal	4	leiomyoma	EMR	NA	NA
Genere et al (15)	78/F	Dysphagia	Upper	20	fibrovascular polyp, duplication cyst	ESD	NA	NA
Yamamoto (16)	72/F	NA	Distal	8	submucosal tumor	ESD	NA	NA
Wang et al (17)	70/M	Abdominal discomfort	Gastroesophageal junction	35	esophageal polyp	EMR	NA	NA
Qin et al (18)	53/M	Abdominal discomfort	Distal	8	NA	EMR	NA	NA
Hua et al (19)	63/M	Abdominal discomfort	Distal	8	Leiomyoma, lymphangioma	ESD	13-72	AWD
Hua et al (19)	65/M	Abdominal discomfort	Distal	12	Leiomyoma, lymphangioma	ESD	13-72	AWD
Hua et al (19)	75/M	Dysphagia	Gastroesophageal junction	15	Leiomyoma, lymphangioma	EMR	13-72	AWD
Our case	83/F	Abdominal discomfort	Gastroesophageal junction	7	fibrovascular polyp	EMR	17	AWD

M, male; F, female; NA, data not available; EP, Endoscopic polypectomy; ESD, endoscopic submucosal dissection; EMR, endoscopic mucosal resection; AWD, alive without disease.

ESGDAs typically have a polypoid appearance, which can be smooth or uneven, with a pedicle or broad base, depending on the stage of the disease. Their sizes range from 3–35 mm, with an average of approximately 10 mm. The cut surface is often greyish-white and solid, and cystic cavity formation can be observed on larger masses. On microscopic examination, the lesions are mainly composed of glandular or cystic cavities of varying sizes. The inner layer of glandular epithelial cells can be multi-layered or protrude into the cavity in the form of papillary hyperplasia, while the outer layer typically consists of basal-layer cells that are fusiform and arranged around the glandular epithelium. Numerous inflammatory cell infiltrates are often observed around the lesions. The inner-layer cells are usually positive for CK7, indicating glandular epithelial differentiation, while the basal layer cells are positive for p63 and CK5/6, indicating myoepithelial and squamous epithelial differentiation. CK20, CDX2, MUC2, MUC5AC, and MUC6 are all negative, suggesting that these glands have no mucus-secreting functions. In addition, atrophy or hyperplasia of the normal acini in the surrounding submucosa is often observed. The Ki-67 proliferation index of ESGDA is usually low, indicating that it is clinically benign.

There are currently no established diagnostic criteria for ESGDA. Based on the previous literature, we believe that the shape meets the following criteria: (1) The lobular structure is composed of many glands or cysts that are mostly double-layered with glandular inner structures. The epithelium and outer layer are composed of basal or myoepithelial cells. (2) The gland or cyst epithelium shows papillary hyperplasia, and the cells have no obvious atypia. (3) Lymphocyte infiltration and lymphoid follicle formation can be seen around the lesion, along with normal acinus atrophy or hyperplasia. Immunohistochemical analysis shows that the outer basal-layer cells express SMA, S-100, and p63 and that the inner glandular epithelium express MUC5B and various CKs, including CK5/6, CK7, CK17, CK18, CK19, and HMWCK. In contrast, CK20, CDX2, MUC5AC, MUC6, and MUC2 markers are all typically negative. In terms of microscopic morphological features, leiomyomas, granulosa cell tumors, fibrous polyps, and lymphangiopathies, which are difficult to differentiate clinically and endoscopically, can be easily distinguished. However, attention should be paid to the identification of esophageal adenocarcinomas and adenoid cystic carcinomas. These can lead to clinical overtreatment, as tissue structure, cell atypia, and invasive boundaries can provide clues, specific immunohistochemical indicators, and high proliferation Ki67 indices.

The treatment of esophageal tumors, especially the surgical methods, has been innovated. Compared with thoracotomy and endoscopy technologies, the intrathoracic robotic-sewn esophageal anastomosis has the same safety and feasibility compared with traditional technologies, and has the characteristics of fast, less bleeding and fast recovery, which is now accepted by medical science (21). However, ESGDAs are benign tumors and endoscopic resection is recommended. Of the 20 cases discussed in this review, all were treated by endoscopic local tumor resection except for two cases with insufficient understanding of the early stages and one case that was accompanied by other epithelial malignant tumors (6). The longest follow-up period was 132 months. The prognosis was favorable, and no recurrence or metastasis was observed. There are several cases of esophageal adenocarcinomas originating from the SMGs been reported, based on the fact that no foci of Barrett’s esophagus or heterotopic gastric mucosa were histologically identified around the carcinoma lesion. However, Considering extremely rare, whether there is a stepwise process from SMGs to adenocarcinoma remains uncertain, the possibility of tumor originated from cardiac gland could not be excluded (22, 23). Due to the relatively rare clinically, whether these are related to the direct carcinogenesis of ESGDA remains to be explored in future studies with large sample sizes and long follow-up periods are necessary to understand the exact behavior of the disease.

## Conclusion

In conclusion, when a polypoid mass is observed in the lower esophagus of an elderly patient under a microscope, where two layers of mild cells form a cystic or papillary shape, the possibility of ESGDA should be considered, and the final diagnosis should be made in combination with immunohistochemical analysis. This will ultimately aid in preventing misdiagnosis or missed diagnosis of ESGDAs. In terms of treatment, ESGDA requires only endoscopic tumor resection.

## Data Availability

The original contributions presented in the study are included in the article/**Supplementary Material**. Further inquiries can be directed to the corresponding author.
